# ‘Surveillance Capitalism, COVID-19 and Social Work’: A Note on Uncertain Future(s)

**DOI:** 10.1093/bjsw/bcab099

**Published:** 2021-06-21

**Authors:** Paul Michael Garrett

**Affiliations:** School of Political Science & Sociology, NUI Galway, Galway, Republic of Ireland

**Keywords:** COVID-19, inequalities, surveillance, surveillance Capitalism, Zuboff

## Abstract

Drawing on Shoshana Zuboff's (2019) *The Age of Surveillance Capitalism*, along with additional sources not ordinarily referenced in the social work literature, the article examines some of the economic and political imperatives that are driving forward new surveillance practices. The aspiration is to provide conceptual coordinates enabling practitioners, educators and those receiving social work services to arrive at a theoretically expansive sense of what may be occurring across a societal canvas. The focus is on a cluster of five enmeshed themes: first, what Zuboff means by ‘surveillance capitalism’; second, why this form of capitalism has appeared so quickly over the past couple of decades; third, what the tech corporations, such as Google, seek to achieve; fourth, how surveillance capitalists aim to eliminate chance by refining technologies so as to try and constitute us as predictable human subjects; fifth, the trajectory of surveillance capitalist interventions and how they are ‘doubling down’ on the processes of data extraction. Zuboff’s book was completed prior to theCOVID-19 global pandemic and, in the latter part of article, it is argued that the current crisis will result in new forms of surveillance becoming socially embedded.

## Introduction

Recent papers in the *British Journal of Social Work* draw attention to the impact of technology (Devlieghere and Gillingham, 2020; Steiner, 2020; Vannier Ducasse, 2020). Significant here has been the exploration of artificial intelligence, machine learning and the increasing use of algorithms that are shaping new—often highly problematic—ways of working (Eubanks, 2017). Although not entirely detachable from this body of literature, the concerns in this article are somewhat more expansive. Here, drawing on Shoshana Zuboff’s (2019) sustained investigation in *The Age of Surveillance Capitalism* along with sources infrequently highlighted in the social work literature, the focus is on some of the main imperatives driving forward new surveillance practices.

First, the article refers to what Zuboff means by ‘surveillance capitalism’; second, it looks at why this form of capitalism has appeared so swiftly over the past couple of decades; third, it examines what the tech corporations, such as Google, seek to achieve; fourth, it explores how surveillance capitalists aspire to eliminate chance by refining technologies so as to try and constitute us as predictable and consuming human subjects; fifth, the main interest is the trajectory of surveillance capitalist interventions and how they are ‘doubling down’ on the processes of data extraction. Exploring such themes is vital because the emerging surveillance technologies discussed by Zuboff are likely to become more extensively deployed within fields such as social work. If, for example, the ‘welfare of the child is the paramount consideration’, as most legal frameworks suggest, why not deploy home surveillance technologies and emotional analytics in the domain of child protection? An argument could be made that such forms of affect recognition, to be discussed later, would better equip practitioners to forensically ‘test’ the veracity of parental statements and dispositions. Here, the argument is not that this should occur, yet one does not have to have a dystopian sci-fi mindset to be able to recognise how surveillance technologies tend to become re-purposed and transfer into areas and practices for which they were not originally intended. For example, electronic surveillance and the related testing for alcohol use and other drugs, formerly used to test prisoners or others in contact with the penal estate, are now used with parents where multi-disciplinary professionals remain unconvinced about their trustfulness (see also BBC News, 2020a). It is not difficult to detect, therefore, how some of the surveillance technologies to be discussed might be eased into the child welfare arena, particularly if the parents concerned come freighted with a symbolic load of potential ‘dangerousness’ (Dale *et al.*, 1986).

Importantly, Zuboff’s book was completed prior to the outbreak of the COVID-19 global pandemic (Malm, 2020). However, as recognised in the final section of the article, much of the content featured in *The Age of Surveillance Capitalism* is of magnified significance because of responses to the pandemic. Engaging with Zuboff’s work today requires her readers to *stretch* her perceptions in order to render them topical and meaningful at this particular conjuncture. The COVID-19 crisis suggests that various new forms of surveillance will be introduced which may become embedded and part of what is presently described, rather too blandly and far too uncritically, as the ‘new normal’ in, and beyond, social work.

Certainly, health surveillance-driven assessments and judgements made by states and their agents are likely to have profound implications on the civil rights of population segments identified as ‘at risk’ or as potentially constituting a ‘risk to others’ (Trottier *et al.*, 2021). Discussions currently taking place in relation to the so-called ‘immunity passports’ may well expand the reach of such pre-occupations (Proctor and Devlin, 2020). What is more, social workers may become a part of public health networks monitoring compliance with how states surveil and regulate mobility. In Italy, for example, the health minister proposed ‘sectioning’ individuals refusing COVID-19-related treatment and it is not unlikely that social services, with a good deal of experience of handling such interventions in the field of mental health, might become incorporated into such schemes (Giuffrida, 2020).

## Surveillance capitalism

### Recognising ‘surveillance capitalism’

Zuboff asserts that ‘surveillance capitalism’ is an ‘antidemocratic and anti-egalitarian juggernaut’ and should be perceived as a ‘market-driven coup from above’ (Zuboff, 2019, p. 513). Its prime, economic imperative is to systematically and relentlessly extract information and data from the activities of ‘users’ of the various ‘services’ it makes available (Zuboff, 2019, p. 87). So, for example, when someone uses Google as a search engine, this produces a ‘behavioural surplus’, which, on account of machine intelligence, can be harvested to predict which products are likely to attract them as consumers. In addition to keywords, each ‘Google search query produces a wake of collateral data such as the number and pattern of search terms, how a query is phrased, spelling, punctuation, dwell times, click patterns, and location’ (Zuboff, 2019, p. 67).

Such information is commodified and traded to companies aiming to better ‘target’ customers. As Zuboff (2019, p. 212) summarises, you ‘get on Google, and it seems like it’s free. It’s not free. You’re giving them information; they sell your information’. This enterprise is, of course, immensely profitable because companies, such as Google and Facebook, are able to mine enormous sets of data. The surveillance capitalists are successful because they have evolved their practices very speedily and indicative of the strategy is, of course, Facebook’s motto, prominently trumpeted within company until 2014, ‘move fast and break things’. According to a UN report, this strategy can be coupled to the ‘importance attached to minimising legal and governmental constraints’ (United Nations General Assembly, 2019, p. 13).

Attentive to the fact that they are actually addressing people’s needs (for communication, access to information and new forms of consumption), surveillance capitalists have also been adept at appropriating a lexicon of, seemingly, benign social work keywords (such as ‘empowerment’, ‘inclusion’, ‘participation’, ‘community’) so as to nurture a particular affective ambiance tapered to entice and retain users for the purposes of data extraction (Zuckerberg, 2017). A good example of how language is manipulated is provided by Amazon where every move by workers is tracked and monitored. If a worker is deemed too slow or is suspected of having taken too much ‘time off task’—abbreviated in companyspeak to TOT—then, they are routinely fired and swiftly replaced. However, Jeff Bezos dubs his gargantuan warehouses ‘fulfilment’ centres (Lecher, 2019). Meanwhile, suggesting that his privately owned corporation has ‘built the world’s largest social infrastructure for collective action’, Zuckerberg (2017) hubristically implies that his corporation is evolving into an alternative UN or even world government. What is also striking in this context is how some of the leading CEOs of ‘surveillance capitalism’ are close to the US Democrats and rhetorically present themselves as, not only ‘cool’ and appropriately philanthropic, but palpably ‘woke’ and achingly ‘liberal’. Nonetheless, whilst skilled at ‘romance and beguile’, the Big Tech sector remains ‘ruthlessly efficient at extinguishing space for democratic deliberation, social debate’ and ‘individual self-determination’ (Zuboff, 2019, p. 193).

### The appearance of ‘surveillance capitalism’

Here, it is vital to recognise that the neo-liberal zeitgeist furnishes a temporal and cultural context for the evolution of ‘surveillance capitalism’ with the main CEOs and associated key players broadly adhering to the key ideological tenets of this particular regime of capital accumulation. During the initial months of the COVID-19 pandemic, the leading surveillance capitalist CEOs experienced an extraordinary wealth ‘surge’. The US-based Institute for Policy Studies (2020, p. 1), for example, reports that between 1 January 2020 and 10 April 2020, thirty-four of the nation’s wealthiest 170 billionaires saw their wealth increase by tens of millions of dollars. The wealth surge of Bezos is ‘unprecedented in modern financial history’ in that his ‘fortune had increased by an estimated $25 billion since January 1, 2020’. At Facebook, Zuckerberg’s personal wealth increased about $22 billion in 2020 (BBC News, 2020b).

In terms of ideology, Zuboff (2019, p. 109) comments on the ‘cyber-libertarian’ philosophy which has driven ‘surveillance capitalism’, and highlights how this is reflected in ‘free speech fundamentalism’ and the notion that governments should not impede, what is presented, as inevitable and simply determined by technological ‘progress’. Relatedly, the suggestion is conveyed that ultimately governmental and public regulations are futile given the dawning of a ‘new age’ (Zuboff, 2019, p. 221). Associated with such tactics is a relentless pursuit and defence of the right to maintain corporate control of cyberspace. Zuboff also adds that the events of 9/11, and the requirements of the US state security apparatus nurtured the burgeoning success of the tech sector in that the government was keen to avail of corporate technical expertise in order to algorithmically identify the potential ‘threats’ posed by ‘radicals’. This big-tech/state nexus is unlikely to change under the Biden administration given that former Facebook personnel occupied strategic positions within his transition team (Scola and Thompson, 2020). Indeed, this team was notably chockful of key figures from Amazon, Airbnb, LinkedIn, Dell, Dropbox and Uber (Birnbaum and Kramer, 2020).

### The aspirations of ‘surveillance capitalists’

Without downplaying the role played of Facebook, perhaps it is Google, created in 1998—and now corporately parented by Alphabet—that is surveillance capitalism’s iconic corporation. Maybe there was a ‘time when you searched Google’, avows Zuboff (2019, p. 261), ‘but now Google searches you’. Although much smaller in terms of the numbers it employs, Google is to surveillance capitalism what the ‘Ford Motor Company and General Motors were to mass-production’ (Zuboff, 2019, p. 63). Significantly, the Mountain View-based corporation ‘recognized the gold dust in the detritus of its interactions with its users and took the trouble to collect it up’ from what is often referred to as the ‘data exhaust’ (Zuboff, 2019, p. 68). Google’s ‘unique prowess’ is its capacity for ‘hunting, capturing, and transforming surplus into predictions for accurate targeting’ (Zuboff, 2019, p. 80). The by-products, or leftovers, of our numerous searches provide raw material which reveal, if analysed, potentially ‘detailed stories about each user—thoughts, feelings, interests—could be constructed from the wake of unstructured signals that trailed every online action’ (Zuboff, 2019, pp. 67–8). This information is a product, which can be sold to companies seeking to sell us a whole array of goods and services. Indeed, Google is best perceived, like Facebook, as a giant ‘advertising platform’ (Srnicek, 2017). The company is also characterised by its alacrity in introducing ‘pricing innovations’, including pricing metrics based on the so-called ‘click-through rates’ or ‘how many times a user clicks on an ad through to the advertiser’s web page, rather than pricing based on the number of views that an ad receives’ (Zuboff, 2019, p. 82).

For capitalism to function, the scale of consumption has to be maintained or else there will be a block in the system. In short, commodities have to be produced, but they also have to be purchased. Hence, there is a need to entice and persuade people to constantly ‘extend the circle of their enjoyments’ (Marx, 1990 [1867], p. 769). What corporations, such as Google, quickly discerned was that the data it accumulated might enable advertisers to ‘deliver a particular message to a particular person at just the moment when it might have a high probability of actually influencing his or her behaviour’ (Zuboff, 2019, pp. 77–8). What is more, after a long decline in the manufacturing sector, the tech industries provided a sluggish capitalism with a new vitality and buoyancy (Srnicek, 2017).

### Creating predictable and consuming human subjects

A fourth component of Zuboff’s analysis is her interrogation of how the surveillance capitalist corporations incessantly seek to eliminate chance by using technology to shape and constitute us as *predictable* human subjects. Central here is the drive to embed certainty and outcomes that are guaranteed on a societal basis. This involves the design of a multiplicity of programmes intent on behaviour modification in order to render our futures entirely predictable; particularly in terms of what we choose to buy and how we relate to each other within the wider fabric of capitalism. For the sake of the ‘plan, the totality of society—every person, object, and process—must be corralled into the supply chains that feed the machines’ (Zuboff, 2019, p. 401).

Expressed somewhat differently, the efforts of surveillance capital are preoccupied with how ‘objects’ act and perform as consumers and workers. One of the primary definers articulating and advocating along these lines is MIT computer scientist and entrepreneur Alex Pentland (2011). Influenced by B. F. Skinner, the American behavioural psychologist and social philosopher, Pentland’s specialism is wearable technology and his perceptions on the role of such devices in the workplace are particularly interesting. His experimental work involving speech recognition technology has, it is maintained, been able to identify ‘profiles of individuals based on the words they use’ and this enables employers and workplace managers to ‘form a team of employees with harmonious social behaviour and skills’ (Zuboff, 2019, pp. 422–3). Pentland also outlines how the information gathered by wearable sensor devices—what he terms ‘sociometers’ or ‘sociometric badges’—is able to measure workplace communications, voice cadences and body language (Choudhury and Pentland, 2003). This might, therefore, empower and assist ‘managers understand who is working with whom and infer the relationships between colleagues’ and ‘would be an efficient way to find people who might work well together’ (Zuboff, 2019, p. 423). According to Pentland, one of the core aims is to address situations where people are not ‘interacting correctly’ and making ‘bad decisions’ (in Berman, 2016). In such scenarios, data can go a long way to mend, what he cryptically terms ‘broken behaviours’ (in Berman, 2016).

It is easy to see how this thinking—and the technology that such thinking spawns—might produce an inflation of surveillance practices within the neo-liberal workplace. It is also manifest that Pentland, and many similar figures in his, see nothing fundamentally wrong with the way that the world is economically configured. Workers, it appears, *should be* exploited in order to maintain current patterns of capital accumulation and the core role of social technicians, such as him, is to assist in the fine tuning of workplace relationships so as to create and maintain a functionally consensual, compliant, profitable workforce (Lecher, 2019). Equally discernible is the more overarching idea that politics should be trumped by social administration and ‘neutral’ governance (see also Buzzi, 2020). Here, the core ideological understanding is that human behaviour ‘must be herded and penned within the parameters of the plan’ (Zuboff, 2019, p. 434). Pentland asserts:

Revolutionary new measurement tools provided by mobile telephones and other digital infrastructures are providing *us* with a God’s eye view of ourselves. For the first time, *we* can precisely map the behaviour of large numbers of people as they go about their daily lives. For society, the hope is that *we* can use this new in-depth understanding of individual behaviour to increase the efficiency and responsiveness of industries and governments. For individuals, the attraction is the possibility of a world where everything is arranged for your convenience—your health check-up is magically scheduled just as you begin to get sick, the bus comes just as you get to the bus stop, and there is never a line of waiting people at city hall (Pentland, 2011, emphasis added).

One might, of course, wonder who exactly helps to constitute the ‘us’ and the ‘we’ overseeing this hubristic and grand vision. What Pentland presents is a smooth-running world, entirely drained of meaningful politics and agonistic encounters, in which social problems are dealt with by coders and associated technicians.

### ‘Doubling down’ on data extraction

A fifth important dimension to Zuboff’s book is her commentary on how surveillance capitalist corporations, such as Google, are aiming to expand the ‘extraction architecture’ to hone ‘prediction products that attract and retain more customers’ (Zuboff, 2019, p. 129). Significant here is the idea, evoked by phrases such as ‘ambient computing’, the ‘internet of things’ and ‘ubiquitous computing’ (Weiser, 1991), that the internet and associated devices may actually disappear by fading into the quotidian background. This notion of ‘disappearance’ is misleading in that what would occur would be an evolving *omnipresence*. In the early 1990s, Mark Weiser (1991, p. 94) suggested that the information and communication technologies of the time might weave themselves into the ‘fabric of everyday life until they are indistinguishable from it’. Subsequently, such notions influenced key players such as Eric Schmidt, the former CEO of Google. In 2015, Schmidt confidently maintained, in the hyperbolic style typical of his sector, that the ‘internet will disappear. There will be so many IP addresses… so many devices, sensors, things that you are wearing, things that you are interacting with, that you won’t even sense it. It will be part of your presence all the time. Imagine you walk into a room and the room is dynamic’ (in Zuboff, 2019, p. 197). The world evoked by this billionaire, in which the internet and connectivity is unshackled from personal computers and smartphones, might appeal to some ‘users’, but the core driver for such aspirations is to maximise the data extraction possibilities in order to create better products for advertisers who can, in turn, target potential customers more efficiently.

As Marx (1981 [1857–58], p. 270) notes, the capital accumulation process is an ‘endless process’ with capital constantly crashing through what have previously been seen as a ‘barrier’. This point, made in the middle of the nineteenth century, adds to Zuboff’s discussion on the expansion of the ‘extraction architecture’ of ‘surveillance capitalism’. Influential figures, such as Schmidt and those sharing his imperatives, hanker to create a new apparatus in which ‘world, self, and body are reduced to the permanent status of objects…His washing machine, her car’s accelerator, and your intestinal flora are collapsed into a single dimension of equivalency as information assets that can be disaggregated, reconstituted, indexed, browsed, manipulated, analysed, reaggregated, predicted, productized, bought, and sold: anywhere, anytime’ (Zuboff, 2019, p. 210).

The creation of this new apparatus is envisaged as occurring on two fronts: by geographically extending operations into ‘real-world’ domains lying beyond the computer or other devices such as the smartphone and by increasingly mining the human body for data (Pentland, 2011). The extension of extraction practices from the ‘virtual’ into the ‘real’ world targets potentially new resources located in the actual, lived environment. For example, Amazon, Google and Apple, in conjunction with Ford and BMW, are exploring ways in which the dashboard of cars can enable shopping from the ‘steering wheel’ (Zuboff, 2019, p. 268). Hence, surveillance capitalists perceive that their future sources of wealth are likely be accrued by accessing ‘new supply routes that extend to real life on the roads, among the trees, throughout the cities’ (Zuboff, 2019, p. 199). Ultimately, the encompassing project is to ‘engineer’ a ‘new nervous system for humanity’ and to evolve a new planetary sensorium (Pentland, 2011). Relatedly, public spaces (such as parks and shopping malls and their inhabitants) will become knowable and readable objects that can be aggregated into a ‘seamless flow of searchable information, sights, and sounds’ (Zuboff, 2019, p. 208).

Although not explored in detail by Zuboff, so-called ‘smart dust’ may well be an area of technological expansion and commercial exploitation in the next few decades. Coined in 1997, the term refers to networks of microelectromechanical sensors, or ‘motes’, which will ‘unlock unprecedented levels of data collection’ (Goldsmith, 2019). These millimetre-sized devices may have the capacity to ‘rove around the world collecting all kinds of data: visual, thermal, chemical and biological’ (Goldsmith, 2019). In short, ‘smart dust is the natural next step for today’s Internet of Things’ (Goldsmith, 2019). However, the deployment of mobile microscopic sensors capable of collecting audio and visual data illuminates a range of questions circulating around the enhanced surveillance capacity of corporations and states.

In the area of health and social care, researchers have also raised the possibility of ‘neural dust’ being implanted in the body and used to monitor the activity of different organs and to stimulate nerves and muscles (Goldsmith, 2019). More generally, tech corporations view the human body as merely another object that can be ‘tracked and calculated for indexing and searching’ (Zuboff, 2019, p. 241). Much of this in taking place under the banner of—another social work keyword—‘personalisation’ (Zuboff, 2019, p. 255). The rendered body could, in fact, furnish another ‘highly lucrative’ behavioural surplus that could be ‘plumbed from intimate patterns of the self’ (Zuboff, 2019, p. 199). Hence, processes of extraction would be targeted at ‘your personality, moods, and emotions, your lies and vulnerabilities’ with every kind of intimacy ‘automatically captured and flattened into a tidal flow of data points’ (Zuboff, 2019, p. 199).

By 2015, 29.5 million US adults used wearable devices—mostly fitness trackers such as Under Armour’s and smart watches (Zuboff, 2019, p. 603). Health insurers, particularly, may become keen to utilise wearable devices to monitor if customers insured are adhering to ‘agreed’ fitness regimes and, of course, evolving health protocols relating to COVID-19 and its possible variants. Digestible sensors might also provide granular data better equipped to detect if there is adequate compliance with prescribed diet and medication. Some may object, of course, to this degree of invasive surveillance, but corporate consultants, such as Deloitte, maintain that inducements to participation might include savings on health insurance premiums. If pricing policies fail to alleviate privacy concerns, insurers are advised to repackage such intensive behavioural monitoring as ‘interactive’ and ‘fun’ (Zuboff, 2019, p. 215).

Bodies can also, of course, be tracked using global position system data. Derived from military intelligence practice, Google is at the forefront of commercial location tracking (Zuboff, 2019, p. 242). Recognising again, the public opposition that this may prompt, the company’s influential chief economist, Hal Varian maintains that all of us can ‘expect to be tracked and monitored, since the advantages, in terms of convenience, safety, and services, will be so great… continuous monitoring will be the norm’ (in Zuboff, 2019, p. 256). Invariably, it is also maintained that the behavioural surplus accruing from this type of tracking and monitoring is only gathered, analysed, retained and stored in such a way as to prevent the identification of individuals. However, informed commentators, such as Zuboff, cast doubt on these assertions given that ‘re-identification science’ reveals just how simple and easy it is to de-anonymise meta-data (Zuboff, 2019, pp. 243–4).

The surveillance capitalist sector appears particularly interested in how the human voice, so central to social work interactions, might increasingly become a source for data extraction. That is to say, there is a realisation that human conversations are a medium which can be harnessed to the extractive apparatus. This realisation can be connected to how private living spaces are increasingly evolving into rich sources of data. Purporting to conjure into being the so-called ‘smart’ houses, home automation systems, such as Amazon Echo and Google Home, ‘render rivers of casual talk from which sophisticated content analyses produce enhanced predictions that “anticipate” your needs’ (Zuboff, 2019, p. 260). This attentiveness to the human voice, or what Zuboff dubs the ‘spoken surplus’, results in analyses of its various components, such as the breadth of vocabulary, along with ‘intonation, cadence, inflection, dialect’ (Zuboff, 2019, p. 261). In 2016, Bloomberg Businessweek declared that Amazon, Apple and Microsoft had commenced a ‘hunt for terabytes of human speech’ (in Zuboff, 2019, p. 262). The same report also gave an account of how Microsoft had constructed ‘mock apartments’ in cities around the globe to record volunteers speaking in typical home settings. The aim was to capture the spontaneous flow of talk, from smartphones and other devices, so as to record, retain and subject to detailed analysis the words used.

As mentioned in the article’s Introduction section, a growing field of data analysis—that may well become more prominent in social work in the future—is variously described as ‘affective computing’, ‘emotion analytics’ and ‘sentiment analysis’ (Zuboff, 2019, p. 281). Zuboff suggests that if this project of ‘surplus from the depths’ is to succeed, then our ‘unconscious—where feelings form before there are words to express them—must be recast as simply one more source of raw-material supply for machine rendition and analysis, all of it for the sake of more-perfect prediction’ (Zuboff, 2019, pp. 281–2). Describing itself as the ‘world’s leading emotion artificial intelligence platform’, a company called Realeyes which is partly EU-funded, deploy specialised ‘software to scour faces, voices, gestures, bodies, and brains, all of it captured by “biometric” and “depth” sensors, often in combination with imperceptibly small, “unobtrusive” cameras’ (Zuboff, 2019, p. 282). The company stresses that measuring emotions can enable those using their software to surpass competitors because often intangible ‘emotions’ can ‘translate into concrete social activity, brand awareness, and profit’ (in Zuboff, 2019, p. 283). Relatedly, the CEO of Affectiva refers to a ‘chip embedded in all things everywhere, running constantly in the background, producing an “emotion pulse’” each time a person checks their phone. She declares: ‘I think in the future we’ll assume that every device just knows how to read your emotions’ (in Zuboff, 2019, p. 288).

These companies are, of course, small in size when compared with the tech giants and we also need to be mindful of the fact that much of this ‘science’ is unproven and that perhaps the publicity speaks, more of the success of entrepreneurs and corporate hucksters in marketing their wares and ‘charms’ (Marx, 1981 [1857–58], p. 287). This discourse is still significant because micro-firms, regarded as potentially profitable innovators, are frequently ‘bought out’ by the large corporations. More fundamentally, the work of companies, such as Realeyes and Affectiva, furnishes us with insights in terms of the trajectory and evolving agendas of the surveillance capitalist sector.

## Surveillance and COVID-19

It is not difficult to detect how some of the concerns, articulated by Zuboff, become extraordinarily magnified in the context of the COVID-19 global pandemic (Milan *et al.*, 2021; Newell, 2021). Prominent, in this context, is the lure—and risk—of tech ‘solutionism’ and the use of advanced forms of surveillance technology to supposedly provide ‘magic bullet’ responses to the spread of the virus. Here the problem is that there is little informed public debate on the ‘solutions’ being considered and deployed. On occasions, it appears that ‘innovation’ talk is distracting public attention from the inequalities which make some people more likely to be lethally infected than others. The disproportionately high number of fatalities in black and minority ethnic communities is a significant issue (Gore, 2020). For example, Public Health England (2020, p. 4) found that the ‘highest age standardised diagnosis rates of COVID-19 per 100,000 population were in people of black ethnic groups (486 in females and 649 in males) and the lowest were in people of white ethnic groups (220 in females and 224 in males)’. This is partly attributable to class and capitalist labour market positioning that leaves black and ethnic minority people more vulnerable; it may also be related to how racism and discrimination detrimentally impacts on engaging with health services.

Questions relating to ‘race’ and racism are largely absent in Zuboff’s book and, indeed, there this is a more pervasive lacuna within the academic field of ‘surveillance studies’. Nonetheless, black bodies are frequently targeted for particular types of oversight and scrutiny (Browne, 2015; Benjamin, 2019; Monahan, 2020, 2021). In the USA, the organisation ‘Data for Black Lives’ (D4BL) expresses concern about how data collected relating to COVID-19 might, given embedded structural racism, become ‘weaponised’ against black and other minority ethnic communities. Although relating to a particular jurisdiction, their concerns have global resonance as does the demand that COVID-19 data should not be used to ‘determine risk. It should not be used to surveil, criminalise, cage, and deny critical benefits’ (Data for Black Lives (D4BL), 2020, p. 26).

Aided by the expertise of the surveillance capitalist corporate sector, states have been able to repurpose habits and ways of relating to the ubiquitous smartphone. For example, in Poland, smartphones have been used to enforce quarantine with individuals mandated to remain at home being compelled to send a photograph of themselves within ‘twenty minutes of receiving a prompt from government officials’ (Aschoff, 2020a); if this does not occur, a police visit is triggered. In this way, the ‘selfie’, perhaps associated with celebratory narcissism, begins to resemble the ‘mugshot’ of the incarcerated prisoner subjected to incessant surveillance. However, utilising algorithms may, in some countries, have beneficially supported contact tracing endeavours and the identification of chains of viral transmission. At the time of writing, the situation is still fluid, but—as the Polish instance illustrates—there are complex human rights and civil liberties issues, focal to the ethics of social work, that need to be addressed (Taylor *et al.*, 2020).

The signatories of a Joint Statement on Contact Tracing (2020) voice concerns that some ‘solutions’ to the crisis may, via ‘mission creep’, result in systems allowing ‘unprecedented surveillance’. More fundamentally, of course, it is not possible to solve the various riddles set by the pandemic merely by ‘coding the perfect app’ (Crocker *et al.*, in Newell, 2021, p. 81). Moreover, the various apps deployed and those being considered for use can too easily be ‘repurposed to enable unwarranted discrimination and surveillance’ (Joint Statement on Contact Tracing, 2020). Facial recognition and biometric identification technology may play a role in relation to the concept of ‘immunity passports’. In the UK, for example, NHSX (the health service’s digital arm) is considering such passports and the ways technology could be deployed (Clarke, 2020a,b). In the USA, smartphones are tracking where people are moving to-and-from during the pandemic (Monahan, 2021). The government has also engaged Palantir, a data mining company founded with seed money from theCIA’s venture capital fund, to produce a new Department of Health and Human Services (HHS) surveillance platform called HHS Protect Now (Aschoff, 2020b). Israel is using its security and intelligence services, honed in its efforts to maintain the illegal presence in the Occupied Territories, to log the movements of those testing positive for COVID-19 (Marciano, 2021). In South Korea, individuals identified as COVID-19 ‘positive’ are publically identified so as to alert others in proximity to them.

In the People’s Republic of China, the Alipay Health Code system assigns individuals differently coloured codes on the basis of COVID-19 status (Chuncheng, 2021; Siqueira Cassiano *et al.*, 2021). The system pivots on the downloading of apps that convert smartphones into, effectively, ‘electronic passes’ (Ling, 2020, p. 14). People wishing to enter public places (e.g. restaurants or boarding trains) must scan a Quick Response (QR) barcode with their smartphones and only those displaying the colour green (‘nothing abnormal detected’) are able to proceed. Other colours result in varying degrees of restriction: yellow (‘quarantine at home’) and ‘red’ (‘quarantine at an approved facility’) (Ling, 2020, p. 14). The specific colour is ‘determined by place-specific algorithms that software engineers tweak daily in line with shifting government directives on city-by-city risk levels’ (Aschoff, 2020b). The system facilitating the tracking of movement is not—at the time of writing—as intensively enforced as it was a number of months ago, but it can be reactivated in the event of new surges in virus infection. This smartphone strategy is complemented by evolving other forms of surveillance technology. For example, a 5G-powered police patrol robots are deployed in public places, such as shopping malls and airports, in large cities such as Guangzhou and Shanghai. These use infra-red thermometers and high-resolution cameras to scan for anyone with a fever or are not wearing a mask. Once detected, they are reported to the police (Aschoff, 2020b).

Whilst recognising the advantages conferred by currently heightened forms of technological monitoring in containing contagion, Harari (2020) ponders if we are at an important ‘watershed in the history of surveillance’. Thus, he invites us to embark on a ‘thought experiment’ so as to consider a:

hypothetical government that demands that every citizen wears a biometric bracelet that monitors body temperature and heart-rate 24 hours a day…The chains of infection could be drastically shortened, and even cut altogether. Such a system could arguably stop the epidemic in its tracks within days…Even when infections from coronavirus are down to zero, some data-hungry governments could argue they needed to keep the biometric surveillance systems in place because they fear a second wave of coronavirus, or because there is a new Ebola strain evolving in central Africa (Harari, 2020).

Certainly, there are a bundle of complex themes emerging with relate directly to social work concerns circulating around human rights, inclusivity and access. For example, if digital methods continued to be deployed and become extended, what of those (frequently older and low income citizens) who lack smartphones? (Broom, 2020). Some states may also decide to incorporate information related to immigration status into an ‘immunity passport’ or QR barcode on a smartphone. Already, it is easy to see how the various surveillance systems that are rapidly coming into use could be easily re-purposed to enhance the surveillance of workers. One area where this is apparent is that associated with wearable sensors that track movement on the ‘shopfloor’ in the manufacturing and logistics sectors. These surveillance technologies, championed by Pentland (2011) and others, were already beginning to proliferate even before the global pandemic. Such devices furnish fine-grained data on the pace that workers are working, who they are associating with, how long they pause to speak to workmates and so on. One does not need to be an unhinged conspiracy theorist to grasp how corporations and states might opportunistically use the crisis to press for more widespread usage in the interests of public health and suppression of COVID-19 or other potential viruses. The danger is that surveillance systems introduced in a time of pandemic risk being permanently retained and repurposed by employers, particularly in the context of a potential economic 'downturn' when the potential revolt of workers needs to be averted and quelled (Aschoff, 2020b). Stretching beyond workplaces, surveillance technologies are also likely to be ramped up to deal with diversity of other protests including those opposing the devastation of the natural environment and male violence (see also Garrett, 2021).

As Harari (2020) avows, many of the supposedly ‘short-term emergency measures will become a fixture of life. That is the nature of emergencies. They fast-forward historical processes’. The dystopian future evoked by Harari is also one that reflects some of the aspirations of Big Tech and the exploitative dynamics that, as we have seen, preoccupy Zuboff. Berardi is a little more optimistic in that he believes that the incessant drift to, for example, constantly surveilled working ‘online’, fast-forwarded by the responses of states and corporations to the pandemic, will prompt dissenters to slowly begin to ‘identify online connectivity with sickness’ and this will result in a craving for ‘experiences that are haptic, shared, void of digital mediation’ (in Petrossiants, 2020). Indeed, such ‘experiences’ might also be perceived as core and defining features of what we have we previously referred to as ‘social work’.

## Conclusion

Whilst not dwelling on social work practice to any large extent, the modest aspiration is of this article has been to provide a number of theoretical coordinates, which might enable practitioners, educators and those receiving social work services to potentially arrive an enlarged sense of what may be taking place. That is to say, the aim has not been to establish deep connections with the micro and quotidian aspects of social work practice; rather the emphasis has been on illuminating a conceptual framework, which has yet to be explored in the social work literature. Others may, of course, wish to build on this analysis to address particular facets of social work that are inflected by the dynamics of neo-liberalised forms of surveillance. Indeed, many years ago, the ground-breaking research of Donna Baines (2004a,b) had already begun to reveal how heightened patterns of electronic surveillance were beginning to be introduced into social work offices (see also Garrett, 2005).

*The Age of Surveillance Capitalism*, the spark for this discussion, is weighed down with conceptual problems, but exploration lies beyond the scope of the article. Zuboff can, however, contribute to our comprehension of what is taking place. Surveillance is not intrinsically repressive and it is vital to recognise that the technologies deployed to monitor and prevent political dissent, for example, can be utilised by those targeted. Human rights activists have used sophisticated tracking and monitoring techniques to try to safeguard the lives of those penetrating hazardous US borders from the Global South (Walsh, 2010). Also in the USA, Darnella Frazier bravely filmed police officer Derek Chauvin extinguishing the life of George Floyd in Minneapolis in 2020 (Belle, 2020). Similarly, Ramsey Orta filmed the police killing of Eric Garner on Staten Island, in New York, during the summer of 2014 (Cooper Jones, 2019).

However, technological innovations are not generally used as instruments intent on facilitating progressive ‘social change and development’ (International Federation of Social Workers [IFSW], 2014). This is because surveillance technologies are steered and channelled by the imperatives of capital and the hegemonic racial and gendered institutional social order that its maintenance demands. Expressed somewhat differently, technology is primarily, but not exclusively, put to work to maximise profit accumulation and to bolster the social and surveillance infrastructure, which enables this to reoccur. Following this logic, we can also grasp how, if the ownership of evolving technologies were socialised, they might immensely benefit people and planet. Potentially, across the spheres of social work and beyond, surveillance technologies might, in fact, aid and support human flourishing.
