# Erratum to: “Surveillance capitalism, COVID-19 and social work: A note on uncertain future(s)”

**DOI:** 10.1093/bjsw/bcab157

**Published:** 2021-08-11

**Authors:** Paul Michael Garrett

*The British Journal of Social Work* (2020). https://doi.org/10.1093/bjsw/bcab099

Some minor updates have been made to this paper to improve clarity and readability. No factual academic content has been changed. The publisher apologises for the errors in the previous version

